# Special Issue: Preterm Birth: Research, Intervention and Developmental Outcomes

**DOI:** 10.3390/ijerph18063169

**Published:** 2021-03-19

**Authors:** Annalisa Guarini, Miguel Pérez Pereira, Anneloes van Baar, Alessandra Sansavini

**Affiliations:** 1 Department of Psychology “Renzo Canestrari”, University of Bologna, 40127 Bologna, Italy; 2 Department of Developmental and Educational Psychology, University of Santiago de Compostela, 15782 Santiago, Spain; miguel.perez.pereira@usc.es; 3 Child and Adolescent Studies, Utrecht University, 3584 CS Utrecht, The Netherlands; A.L.vanBaar@uu.nl

As indicated by the World Health Organization, preterm birth is a relevant public health issue, being one of the leading causes of death in children under five years of age. Its incidence has been increasing in most countries, with inequalities in terms of survival rates and negative outcomes around the world [1,2]. Preterm birth is a critical event that affects not only child development, but also families and societies, particularly in the care and educational settings [3].

The articles included in this Special Issue will add new considerations on prematurity, focusing on: (a) the effect of preterm birth on developmental outcomes from early life to adulthood, taking into account risk and protective factors; (b) the role of parenting and social environment; and (c) interventions that can promote the well-being of preterm children and their families.

The first section of articles explores the effect of preterm birth on developmental outcomes from infancy to adulthood. Some articles reveal that preterm participants show significant differences with respect to normative values and control groups. A higher incidence of learning impairments in very preterm children (gestational age < 33 weeks), compared to normative values, is described at the end of primary school [4]. Delays in very preterm samples (<32 weeks and very low birth weight < 1500 g) also persist into adulthood [5]. Indeed, preterm adults show lower motor and IQ scores compared to full-term adults and adult motor outcomes are predicted by early motor difficulties [5]. A systematic review [6] describes persistent mild difficulties in executive function, short-term verbal memory, literacy, attention skills and processing speed, revealing suboptimal outcomes in late preterm children (34–36 weeks of gestation). In contrast, other studies suggest that the differences between preterm and full-term samples are not revealed by group comparisons but are instead detected by analysing the interplay between risk and protective factors. A first study finds that even if the preterm and full-term groups do not differ in parenting stress level and mind-mindedness (the caregivers’ representation of a child’s mental life), the mothers of preterm infants with higher stress show more non-attuned comments with their children [7]. In a second study, preterm (<37 weeks of gestation) and full-term late talkers do not differ in child language measures and parental speech input at 30 months [8]. However, child and parent risk and protective factors explain the interindividual variability in children’s spontaneous speech and reported language measures, which reveals a mutual influence between the late talkers’ speech and the quality of the parents’ speech input [8]. Another study finds that healthy preterm children do not show cognitive delay at 22 and 60 months, even when including children with different degrees of immaturity [9]. In contrast, cognitive development is moderated by biological factors and the quality of the home environment [9].

These studies confirm the need to overcome the notion of impaired versus intact domains suggesting that, as proposed for other populations with atypical development, scores in the normal range or the absence of differences between preterm and control groups do not necessarily provide evidence of typical developmental trajectories [10]. In addition, these studies highlight the importance of follow-up programs with standardised and repeated assessments after discharge, in agreement with the European Standards of Care for Newborn Health [11]. As suggested by the papers in this Special Issue, to detect early risks and minimize negative impacts, there is a need to include late preterm children in follow-up programs, as well as to extend standardised assessment beyond the first years of primary school.

A second section of the studies analyses the role of parenting and social environment in modulating the effect of preterm birth on developmental outcomes. Concerning parenting, a first study analyses the complex relationship among gestational age (32–41 weeks of gestation), attention capacities, and maternal sensitivity at 18 months and receptive and expressive language at 24 months, revealing an effect of gestational age on receptive language, both direct and indirect, through the child’s alerting attention, and a direct effect of maternal sensitivity on expressive language [12]. A second study analyses whether parenting behaviour at 18 months can mediate the association between birth status and child problems at six years [13]. The mediation of the parenting behaviour is not confirmed, as preterm birth predicts less limit-setting in mother–child interaction at 18 months and child attention problems at six years, while maternal structure at 18 months predicts fewer internalizing and externalizing problems in both preterm and full-term children [13]. Other studies investigate the role of the social environment on preterm delivery and child development. A strong association between maternal mental illness and preterm delivery is found in socioeconomically disadvantaged areas of inland Southern California [14]. In addition, older mothers, non-Hispanic Black mothers, and mothers with Medicaid or other insurance status have a higher risk of preterm delivery [14]. Another study describes a developmental disadvantage at 24 months in preterm infants born in Italy from migrant mothers, originating from less-developed countries (i.e., Pakistan, Côte d’Ivoire, Cameroon, Nigeria, Eritrea, Senegal, Ethiopia) and exclusively fed formula milk [15]. This study confirmed the benefit of human milk that should be the first choice for preterm newborns [1]. However, when this is impossible, an intact protein preterm formula, compared to an extensively hydrolysed infant formula, is associated with a shorter time to full enteral feeding and higher achieved feeding volume in preterm infants [16].

The studies grouped in this second section stress the importance of identifying the factors that may mediate the effect of preterm birth on developmental outcomes, opening up new research challenges on possible cascading effects in the preterm population [3]. In addition, they point out the need to increase targeted prevention among vulnerable populations, confirming that more knowledge is needed to understand how to prevent preterm birth as well as its negative consequences [1].

The last section includes two articles that describe interventions to support families and promote the child’s skills. Preterm birth has an important impact on parents and families, especially when it occurs before 28 weeks of gestational age. Indeed, in the first year at home, the parents of children born in Sweden report prolonged worries for the child and their own emotional state, and they appreciated a post-discharge home-visiting program to support parent–child interaction and encourage well-being for parents and children [17]. A second study analyses the effect of a parent dialogic book reading intervention in a sample of late talkers, revealing the efficacy of the intervention in improving expressive lexical and syntactic skills in both full-term and low-risk preterm children (<37 weeks of gestation) between two and three years of age [18]. Concerning low-risk preterm late talkers, the intervention impacts mainly on their emergent syntactic skills [18].

The promising results of both studies suggest the relevance of proposing short, ecological and cost-effective programs for preterm children and their families in order to enrich their environment, modify constraints, and increase protective factors [19].

In conclusion, this Special Issue confirms that an interdisciplinary approach is mandatory to understand the complexity of the risk factors affecting preterm birth and to promote protective factors in order to support the families and children’s developmental trajectories. Only by integrating the results of studies carried out in collaboration among different professional areas of expertise, including paediatricians, psychologists, therapists, educators and teachers, can we improve the care of preterm children by taking into account their family and educational context. Secondly, multiple assessment points, integrating standardised assessment and observation in ecological contexts and emphasizing longitudinal design and multimethod approaches are the best way to grasp the complexity of the phenomenon. Finally, this Special Issue addresses the need to support vulnerable populations, improve follow-up programmes, and develop new interventions to promote the well-being of preterm born children and their families.
